# Reflections on a national public health emergency response to carbapenemase-producing Enterobacterales (CPE)

**DOI:** 10.1017/S0950268822000218

**Published:** 2022-03-18

**Authors:** Hilary Humphreys, Martin Cormican, Wendy Brennan, Karen Burns, Diarmuid O'Donovan, Therese Dalchan, Shirley Keane, Anne Sheahan

**Affiliations:** 1 Department of Clinical Microbiology, Royal College of Surgeons in Ireland, Dublin, Ireland; 2 Department of Microbiology, Beaumont Hospital, Dublin, Ireland; 3 Antimicrobial Resistance and Infection Control Team, Health Service Executive, Dublin, Ireland; 4 Department of Bacteriology, School of Medicine, National University of Ireland Galway, Galway, Ireland; 5 Carbapenemase-producing Enterobacterales Reference Laboratory, Galway University Hospital, Galway, Ireland; 6 Centre for Public Health, School of Medicine, Dentistry and Biomedical Sciences, Queen's University Belfast, Belfast, UK

**Keywords:** Carbapenemase-producing Enterobacterales, endemic, environmental reservoirs, national strategy, screening, surveillance

## Abstract

Carbapenemase-producing Enterobacterales (CPE) are important globally. In 2017, Ireland declared a national public health emergency to address CPE in acute hospitals. A National Public Health Emergency Team and an expert advisory group (EAG) were established. The EAG has identified key learnings to inform future strategies. First, there is still an opportunity to prevent CPE becoming endemic. Second, damp environmental reservoirs in hospitals are inadequately controlled. Third, antibiotic stewardship remains important in control. Finally, there is no current requirement to extend screening to detect CPE outside of acute hospitals. These conclusions and their implications may also be relevant in other countries.

## Background

The Enterobacterales include bacteria such as *Escherichia coli* and *Klebsiella pneumoniae*. They are normal inhabitants of the gastrointestinal tract, but can cause serious infections, including bloodstream infection (BSI). Carbapenems are broad-spectrum beta-lactam antimicrobials used to treat Enterobacterales infections, resistant to third-generation cephalosporins and beta-lactam/beta-lactamase inhibitor combinations. Carbapenem-resistant Enterobacterales (CRE) are increasingly important, especially those mediated by carbapenemases (i.e. carbapenemase-producing Enterobacterales (CPE)) because these enzymes are encoded on mobile genetic elements that spread amongst different Enterobacterales [1]. These carbapenemases include *K. pneumonia*e carbapenemase (KPC), oxacillinase 48 (OXA-48) and the metallo-beta-lactamases, such as New Delhi metallo-beta-lactamase (NDM), with potential for widespread dissemination [2].

Because of delays in detection and starting appropriate treatment, those infected with CPE may be adversely affected. In an intensive care unit study, colonisation independently predicted CRE infection [3]. A multi-centre study found that carbapenem resistance was associated with an increased length of hospital stay and in-hospital mortality [4].

Key infection prevention and control (IPC) strategies include building core skills (e.g. behavioural changes, hand hygiene and cleaning), capacity and resources (e.g. staffing), the investigation of outbreaks, isolation/cohorting of positive patients and antimicrobial stewardship [5]. Strengthening laboratory diagnostics and increasing screening volumes are important as CPE detection is more challenging than for other multi-drug-resistant microorganisms, such as methicillin-resistant *Staphylococcus aureus* (MRSA). There is increasing emphasis on environmental reservoirs, e.g. hospital sinks, showers, previously well recognised for other Gram-negative bacilli such as *Pseudomonas aeruginosa* [6].

In Ireland, the Minister for Health declared a National Public Health Emergency (NPHE) on CPE in October 2017, as Ireland's first National Action Plan on Antimicrobial Resistance (iNAP) was being launched [7]. A NPHE Team (NPHET) was convened to focus efforts on improving resource provision (national and local), surveillance, enhancing communication, increased testing and the development of policies and guidance to limit dissemination. A CPE expert advisory group (EAG) was convened to provide evidence-based guidance to the NPHET. This EAG first met in December 2017 and reviewed surveillance data, responded to enquiries on preventative aspects and reviewed draft clinical guidelines, i.e. for acute hospitals and specific services, such as haemodialysis. It has also supported the NPHET in securing additional funding (€24.4 million with 301 whole time equivalents posts in 2018–2021). It has also reviewed the evolving epidemiology to inform national strategy and ensure alignment with the publicly funded healthcare service, the Health Service Executive's (HSE) antimicrobial resistance and infection control (AMRIC) implementation plan. An interim analysis of the effectiveness of that NPHE process has been recently reported [8]. Governance structures were established within the HSE to oversee and direct the establishment of a CPE Implementation Team (later incorporated into AMRIC Oversight and Implementation Teams) who were tasked with the implementation of guidance agreed by the CPE NPHET and EAG (e.g. implementation of CPE screening programme).

## Epidemiology of CPE in Ireland

In 2011, CRE was made a notifiable infection in Ireland. In 2012, the National Carbapenemase-Producing Enterobacterales Reference Laboratory (NCPERL) was established. These decisions assisted in assessing the true extent of CPE. The declaration of an NPHE has given greater impetus to more widespread surveillance and improved IPC. Surveillance resulted in increased reporting of clusters and outbreaks. Although one of the best-characterised outbreaks was caused by NDM carbapenemase, CPE mediated by OXA-48 has been more common [9]. In one prevalence survey, 1.38% of in-patients were colonised, all with OXA-48 [10]. While 65% of CPE in Ireland in 2018 was from hospital inpatients and 87% from screening specimens, CPE has also been detected from patients attending general practitioners. In addition, CPE has been detected in aquatic environments, including hospital effluent, a potential route for wider dissemination [11].

**Fig. 1.**
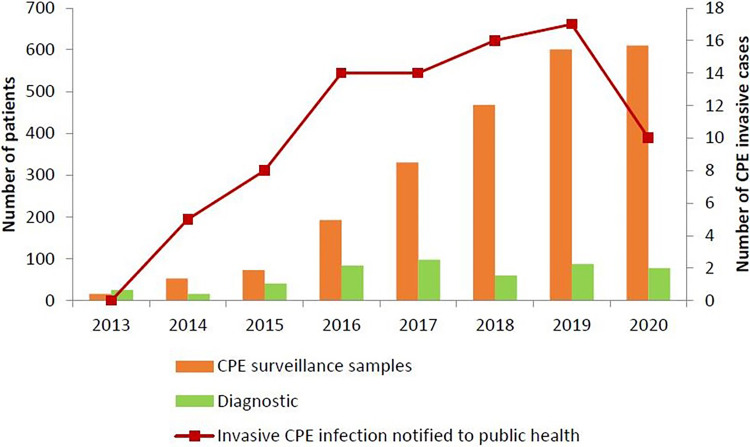
Newly detected patients with CPE by year and invasive CPE infections (https://www.hpsc.ie/a-z/microbiologyantimicrobialresistance/strategyforthecontrolofantimicrobialresistanceinirelandsari/carbapenemresistantenterobacteriaceaecre/surveillanceofcpeinireland/cpeannualreports/CPE%20Enhanced%20Surveillance%20Report%202018.pdf) (Accessed 21 May 2021).

The latest annual summary data to the end of 2020 is outlined in Figure 1. Active surveillance maintained through the coronavirus disease 2019 (COVID-19) pandemic demonstrates a low prevalence. Monthly updates to the end of September 2021 are available at https://www.hpsc.ie/a-z/microbiologyantimicrobialresistance/strategyforthecontrolofantimicrobialresistanceinirelandsari/carbapenemresistantenterobacteriaceaecre/surveillanceofcpeinireland/cpemonthlysurveillancereports/AMRIC%20September%20%202021%20CPE%20monthly%20report-MC-Agreed.pdf.

The data show a stable level of acquisition of CPE colonisation and ongoing detection from hospital environments where sampling is performed. Reference laboratory data show that the CPE genotype detected in hospital environments generally corresponds to that genotype acquired in that hospital (data not shown).

## Ongoing IPC issues

Here, we reflect on a series of questions considered by the EAG to address ongoing issues and what needed to be done next (Table 1).

**Table 1.** Questions considered by the CPE EAG

Question	Relevance
Is CPE endemic in Ireland?	No. If generally endemic in acute hospitals, eradication may not be possible.
Is CPE acquisition still primarily associated with acute hospitals?	Yes. This informs screening and IPC strategies, i.e. continuing to focus on spread in acute hospitals
What is the contribution of persistent hospital environmental reservoirs?	This is a key factor, and therefore preventing person-to- person transmission may not be sufficient for control/eradication.
Should we increase the emphasis on reducing the risk from persistent reservoirs?	Yes. We need to sample appropriately and take measures to reduce spread from such reservoirs.
What is the role of antimicrobial stewardship in controlling CPE acquisition?	Limiting the use of antimicrobial agents may reduce acquisition, excretion and persistence in the hospital environment.
Does community acquisition and spread justify extending screening to the community?	No. Community acquisition is not currently a key factor and screening in the community is therefore not warranted.

CPE, carbapenemase-producing Enterobacterales; IPC, infection prevention and control.

### Is CPE endemic in Ireland?

No. The declaration of an NPHE led to a structured programme of increased testing on acute hospital admission, based on national guidelines (https://www.hpsc.ie/a-z/microbiologyantimicrobialresistance/strategyforthecontrolofantimicrobialresistanceinirelandsari/carbapenemresistantenterobacteriaceaecre/guidanceandpublications/Control%20of%20CPE%20in%20the%20acute%20hospital%20setting.pdf), monthly reporting of CPE and an assessment of laboratory capacity. All new CPE are submitted to the NCPERL for characterisation.

The term ‘endemic’ is classically applied to an infection. Here, it is appropriate to include CPE infection and colonisation. An endemic state implies that the condition persists at a baseline level in an area in the absence of continued new introduction.

The rate of new detections for CPE appears to have reached a relatively stable plateau (approximately 15 per 100 000 population per year for 2019 and 2020, with 709 and 705 new detections, respectively, in a population of 4.9 million). The great majority has been in people with no history of travel outside Ireland, e.g. the index case in the first report of NDM CPE was admitted to hospital from the community [9]. Particular carbapenemases, such as KPC or OXA-48 and specific OXA-48 plasmids have persisted in some hospitals for over a decade [12]. While CPE is endemic in some hospitals that report monthly new acquisitions, most do not show this pattern. Although outbreaks in community long-term care facilities have occurred, there are no reports of persistent foci of acquisition. Therefore, it is not currently endemic in the community and hence there may be an opportunity to prevent it becoming endemic throughout the healthcare system.

### Is CPE acquisition still primarily associated with acute hospitals?

Yes. CPE acquisition without associated in-patient hospital stay is relatively uncommon in Ireland. However, CPE has been detected in the partners of people with CPE, suggesting that the household transmission of CPE can occur.

CPE has been detected in hospital effluent, sewage and seawater, emphasising the risk that CPE could disseminate into the wider environment from hospitals. However, in contrast to extended spectrum beta lactamase (ESBL)-producing Enterobacterales, CPE is currently not as frequently detected in water outlets and sewage [13, 14]. This suggests that effective control or eradication may still be possible, as the main challenge is in hospitals.

### What is the contribution of persistent hospital environmental reservoirs?

The enhanced focus on screening for asymptomatic carriage of CPE and contact precautions is likely to have limited person-to-person transmission. It is difficult to quantify the proportion of CPE acquired from person-to-person transmission, compared with persistent environmental reservoirs. However, based on isolates submitted to the NCPERL, the detection in damp areas (e.g. showers, sinks) is common in hospitals with persistent acquisition. The extent of this was not apparent until the widespread adoption of national guidance on sampling. Based on the match between environmental and patient isolates, we consider that acquisition from persistent environmental reservoirs is now a key factor in hospital acquisition. Therefore, an effective approach to manage the risk from persistent environmental reservoirs is needed.

### Should we increase the emphasis on reducing the risk from persistent reservoirs?

Yes. In Ireland and elsewhere, CPE has been detected from showers, sinks and patient toilets in hospitals [6, 11]. Managing this requires the optimisation of infrastructure, including the installation of modern sanitary ware that is more appropriate for hospital settings, and effective cleaning/decontamination. Hospitals with ongoing transmission need to identify transmission hot spots with appropriate environmental testing. Our experience is that detection in the environment is critically dependent on sampling and testing methods. Sampling the hospital environment with a standard diagnostic swab fails to detect contamination that is revealed with more rigorous methods recommended by the CPE EAG (https://www.hse.ie/eng/about/who/healthwellbeing/our-priority-programmes/hcai/resources/cpe/environmental-testing-for-carbapenemase-producing-enterobacterales.pdf). In the absence of suitable sampling methods, the extent of environmental contamination is likely to be underestimated. New approaches to reduce the level of CPE in drains and/or back wash from drains to touched surfaces, may also be required.

### What is the role of antimicrobial stewardship in controlling CPE acquisition?

Exposure to broad-spectrum antimicrobial agents is a risk factor for CPE acquisition [15]. Antimicrobials probably select for CPE colonisation if a patient is exposed to CPE, and are likely to increase shedding of CPE in the faeces of colonised patients. Antimicrobials in urine and faeces may contribute to sustaining CPE in hospital drainage systems. Avoiding unnecessary antimicrobials and minimising the use of broad-spectrum agents are likely to be important in controlling CPE, and may reduce the risk of CPE gaining access to and persisting in hospitals.

### Does community acquisition and spread justify extending screening to the community?

No. The greatest risk of transmission with adverse consequences, e.g. BSI, occurs in acute hospitals. Hence, this is where testing for carriage is most important. While there may be CPE carriers in the community, the risks of transmission and infection there are probably low. Furthermore, increasing the numbers tested on hospital admission will detect such patients. Therefore, currently there is no requirement to extend screening for CPE carriage outside of acute hospitals in Ireland, e.g. in nursing homes and other residential care units.

## Next steps

Guidelines on CPE/CRE understandably focus on acute care [5, 16]. National priorities on controlling multi-drug-resistant bacteria will vary by country, epidemiology and the political priority it is accorded (https://www.gov.uk/government/publications/g7-health-ministers-meeting-june-2021-communique/g7-health-ministers-meeting-communique-oxford-4-june-2021).

In Israel in the mid-2000s, a national strategy was implemented to control CPE in response to large numbers of cases [17]. This addressed testing, specific nursing staff assigned to CPE positive patients. A reduction in new acquisitions followed, prompting reform of national infrastructure for IPC, surveillance of healthcare-associated infections (HCAIs), the strengthening of reference laboratories, a national programme for antibiotic stewardship and a focus on antimicrobial agents in agriculture.

Most measures to contain CRE/CPE are generic, and apply to other HCAIs. Furthermore, as in Ireland, the declaration of an NPHE around CPE has supported and will continue to help co-ordinate investment in building IPC capacity and infrastructure, especially in hospitals with older buildings and outdated physical infrastructure.

Failure to control CPE has significant financial consequences. An economic evaluation of a CPE outbreak in London in 2014–2015 estimated costs at €1.33 million, the largest cost being reduced capacity for elective procedures due to bed closures [18]. A simulation of a toolkit from the US Centers for Disease Control and Prevention found that cost savings were greatest if all hospitals implemented a regional coordinated approach [19]. Hence, such an approach across hospitals and applied nationally where possible, is most likely to be effective, and is a prudent use of resources.

The declaration of the CPE NPHE, reflecting a strong policy commitment In Ireland, has seen a greater emphasis on the prevention of HCAIs and enhanced IPC measures. This national approach with associated governance arrangements, performance measurements, management systems and structures has supported and informed the establishment of an NPHET to address the COVID-19 pandemic in Ireland. This has resulted in a coordinated and multi-agency approach, with many positive consequences. This was not the case in Ireland when MRSA or ESBL-producing Enterobacterales emerged some decades ago, with both becoming irreversibly endemic in the country.

Following the NPHE response, CPE acquisitions have stabilised [8] (and Fig. 1). This reflects progress in reducing person-to-person acquisition through the detection of colonised individuals and the implementation of transmission-based precautions. However, surveillance for acquisition, environmental sampling and molecular characterisation of isolates has suggested a key role for persistent environmental reservoirs in hospitals. These have likely contributed to failures to eradicate CPE acquisition in some hospitals, notwithstanding rigorous measures to reduce person-to-person transmission. Therefore, measures to manage person-to-person transmission, must be accompanied by a focus on environmental monitoring and control, to drive the transition from stable levels to progressive reduction and even eradication.

## Data Availability

Much if not most of the data described is publicly available. However, we are happy to facilitate researchers, healthcare professionals and others in accessing other data and information on enquiry with reasonable requests.
